# Primary Pleural Hydatidosis—A Rare Occurrence: A Case Report and Literature Review

**DOI:** 10.3390/medicina56110567

**Published:** 2020-10-27

**Authors:** Cornel Savu, Alexandru Melinte, Vasile Grigorie, Laura Iliescu, Camelia Diaconu, Mihai Dimitriu, Bogdan Socea, Ovidiu Stiru, Valentin Varlas, Carmen Savu, Irina Balescu, Nicolae Bacalbasa

**Affiliations:** 1 Department of Thoracic Surgery, “Marius Nasta” National Institute of Pneumophtisiology, 050152 Bucharest, Romania; drsavu25@yahoo.com (C.S.); alexandru.melinte@gmail.ro (A.M.); vasile.grigorie@gmail.ro (V.G.); 2 Internal Medicine Department, “Carol Davila” University of Medicine and Pharmacy, 020021 Bucharest, Romania; laura.iliescu@gmail.ro (L.I.); drcameliadiaconu@gmail.com (C.D.); mihai.dimitriu@gmail.ro (M.D.); ovidiu.stiru@gmail.ro (O.S.); valentine.varlas@gmail.ro (V.V.); 3 Department of Internal Medicine, “Fundeni” Clinical Institute, 022328 Bucharest, Romania; 4 Department of Internal Medicine, Clinical Emergency Hospital of Bucharest, 105402 Bucharest, Romania; 5 Department of Obstetrics and Gynecology, “Sf. Pantelimon” Emergency Clinical Hospital, 021661 Bucharest, Romania; 6 Department of Surgery, “Sf. Pantelimon” Clinical Hospital, 021661 Bucharest, Romania; bogdan.socea@gmail.ro; 7 Department of Cardiovascular Surgery, “Prof. Dr. C.C. Iliescu” Emergency Institute for Cardiovascular Diseases, 022322 Bucharest, Romania; 8 Department of Obstetrics and Gynecology, “Filantropia” Hospital, 011171 Bucharest, Romania; 9 Department of Anesthesiology, “Fundeni” Clinical Institute, 022328 Bucharest, Romania; carmen.savu@gmail.ro; 10 Department of Visceral Surgery, “Ponderas” Academic Hospital, Bucharest, 021188 Bucharest, Romania; irina.balescu@ponderas-ah.ro; 11 Center of Excellence in Translational Medicine, Department of Visceral Surgery, “Fundeni” Clinical Institute, 022328 Bucharest, Romania

**Keywords:** primary, pleural, hydatidosis, Albendazole, echinoccocus

## Abstract

*Introduction*: The larvae of Echinococcus, a parasitic tapeworm, cause hydatid disease. The most commonly involved organ after the liver is the lung but there are cases of hydatid cysts in all systems and organs, such as brain, muscle tissue, adrenal glands, mediastinum and pleural cavity. Extra-pulmonary intrathoracic hydatidosis can be a diagnostic challenge and a plain chest x-ray can be misleading. It can also lead to severe complications such as anaphylactic shock or tension pneumothorax. The purpose of this paper is to present a severe case of primary pleural hydatidosis, as well as discussing the difficulties that come with it during diagnosis and treatment. *Case Report:* We present the case of a 43-year-old male, working as a shepherd, presenting with moderate dyspnea, chest pain and weight loss. Chest x-ray revealed an uncharacteristic massive right pleural effusion and thoracic computed tomography (CT) confirmed it, as well as revealing multiple cystic formations of various sizes and liquid density within the pleural fluid. Blood work confirmed our suspicion of pleural hydatidosis with an elevated eosinophil count, typical in parasite diseases. Surgery was performed by right lateral thoracotomy and consisted of removal of the hydatid fluid and cysts found in the pleura. Patient was discharged 13 days postoperative with Albendazole treatment. *Conclusion:* Cases of primary pleural hydatidosis are very rare but must be taken into consideration in patients from endemic regions with jobs that may have exposure to this parasite. Proper treatment, both surgical and antiparasitic medication, can lead to a full recovery and a low chance of recurrent disease.

## 1. Introduction

Hydatidosis is a parasitic disease caused by the Echinococcus larvae. There are four species of worms responsible for the presence of this disease in humans; however, the most frequent ones that cause cystic echinococcosis are granulosus, 95% of cases, and multilocularis. Echinococcus is a zoonotic disease requiring two mammals, one intermediate host (sheep or cattle) and the definitive host in dogs, wolves or foxes [1].

Batsch first described the form of Echinoccocus granulosus in 1786 and the first case described in the literature of a hydatid cyst is attributed to Bremser in 1821 [2]. In 1908, Rudolphi published a parasitology treaty where the term of hydatid cyst was first used [3]. In most cases, the primary localization of hydatid cysts is in the liver (60–80% of cases) with the lung being the second most common location (10–30%). In the remaining 10–15% of cases, either by haematogenous or lymphatic dissemination or through the veno-venous anastomosis of the Retzius space, the parasite can be found in any organ, tissue or cavity [4]. Primary pleural hydatid cyst is a very rare occurrence and is most often solitary [5].

Human contamination occurs by ingestion of parasite eggs by contaminated food, water or direct contact with the host. Even if hydatid cysts can develop anywhere in the body, liver and lung development are the most common. Pleural hydatidosis is a very rare disease, most cases being secondary to peripheral lung cysts that rupture or herniate in the pleural cavity.

Primary pleural hydatid cysts fall under the category of extra-pulmonary intrathoracic cysts, alongside those found in the parietal pleura, mediastinum, pericardium, diaphragm, fissures and chest wall, by either lymphatic or hematogenous dissemination.

## 2. Case Presentation

**Figure 1:**
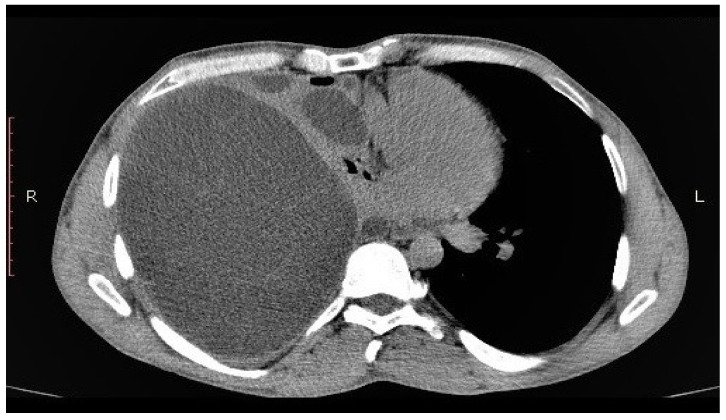
Computed tomography (CT) view of primary pleural hydatid disease.

A 43-year-old male came into our department complaining of chest pain, dyspnea, persistent cough and weight loss; symptoms appeared during the last three months. From the patient history, we found that he was a heavy smoker, occasional consumer of ethanol as well as working with livestock as a shepherd. A routine chest x-ray showed a massive pleural effusion in the right hemithorax, with multiple round-shaped opacities within it. Blood work revealed an elevated eosinophil level (10%) with no other modified parameters. To confirm our suspicion of hydatid disease, we performed a thoracic CT scan, which revealed a large pleural effusion with multiple cystic formations of varying sizes within it (Figure 1).

**Figure 2:**
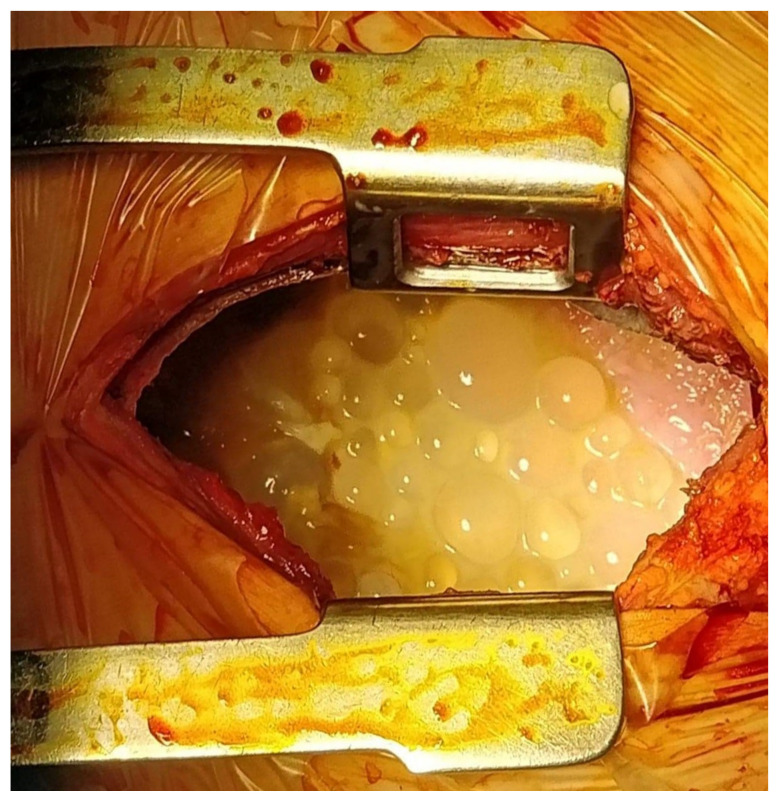
Intraoperative view of primary pleural hydatidosis.

After complete investigation of the patient, we started preoperative antiparasitic treatment with Albendazole 15 mg/kg/day for 6 days and then surgery was performed by using a lateral thoracotomy through the fifth intercostal space. After opening the pleural cavity, we introduced a hypertonic saline solution as well as oxygenated water 10% in order to inactivate the scolices. After draining approximately 4 L of ivory-colored fluid from the pleural cavity we discovered several hundred hydatid cysts with sizes ranging from 1–2 mm to 5–6 cm and the right lung was collapsed in the hilum. Cysts were round, well defined with a clear liquid content. Besides the intact cysts, we also found several ruptured ones, which evacuated and further contaminated the pleural cavity (Figure 2).

After removing all the cysts from the pleural space, we performed several more pleural lavages using a hypertonic saline solution as well as an iodine solution. Further inspection revealed no other cysts in the lung, pleura, diaphragm, mediastinum or pericardium. In addition, there was no sign of rupture or migration of the cyst from any other thoracic organ, leading us to conclude that it was a primary pleural hydatidosis. Before closing, we inflated the lung and there was no air leakage and no other visible lesions on the parenchyma. We placed a single chest drain and the thoracotomy was closed in layers.

Parasitological tests performed on the pleural liquid as well as the parasitic material removed during surgery confirmed the presence of Echinoccocus. Additionally, IGG-specific ELISA tests performed from the pleural liquid confirmed the same.

Chest x-ray performed first day postoperative showed an almost fully expanded lung with no fluid or pneumothorax and a pleural drainage of approximatively 600 mL. The patient also received antiparasitic treatment with Albendazole 15 mg/kg/day. The patient spent 2 days in the intensive care unit (ICU) and was discharged on the 13th postoperative day with the indication of continuing Albendazole treatment for 1 year with 15 mg/kg/day due to the severity of the disease.

Follow-up showed no signs of recurrence with a normal chest x-ray and an improved lung volume function at one month, 6 months and 1 year.

## 3. Discussion

Larvae of the Echinoccocus parasite cause hydatid disease and humans are an accidental host either from consuming contaminated meat or from unwashed fruits and vegetables [1].

Dévé published the first significant study on secondary pleural hydatid disease in 1937 and his conclusions were that all pleural presentations of hydatidosis were secondary to either a lung or liver dome cyst that ruptured into the pleural space, stating that primary pleural hydatidosis does not exist [6]. However, future case reports, such as that of Rakower published in 1964, point to the existence of this pathology [7].

Although most hydatic cysts form in the liver or the lung, due to the possibility of haematogenous dissemination, lesions can develop anywhere in the body. Most common cause of pleural hydatidosis is by either trans-diaphragmatic contamination from cysts located in the right upper hepatic lobe or from the rupture of a peripheral lung cyst [8].

Primary pleural hydatid cysts fall under the category of extra-pulmonary intrathoracic cysts, alongside those found in the parietal pleura, mediastinum, pericardium, diaphragm, fissures and chest wall. Primary pleural hydatidosis manifests as the presence of either a solitary pleural hydatid cyst or as a parasitic pleural effusion [5]. Massive pleural effusions are usually of malignant origin (58% of cases), parapneumonic (23%), tuberculosis (10%), with the remaining cases having diverse origins [9]. Parasitic pleural effusion developed in the case of Echinoccocus is more frequently found as a complication of either a pulmonary hydatid cyst that ruptures in the pleura or as a liver hydatid cyst with intrathoracic development. Parasitic pleural effusion is extremely rare and unusual in medical practice [10].

Primary pleural hydatidosis is found in less than 1% of hydatidosis cases [11]. In our case, we could not find any lesions of the pulmonary parenchyma or any other thoracic lesion. Due to this, we concluded that in our case the diagnosis was that of primary pleural hydatidosis. Although not frequent, primary pleural hydatidosis has been presented as an exceptional situation for hepatic hydatidosis [12,13]. Primary development of a hydatid cyst within the parietal pleural structures is possible and can later lead to hydatid pleural effusion. The histological structure of the cystic membranes allows the passage of calcium, magnesium, water, urea as well as other nutritional substances that may pass through by diffusion and favor the development of the cyst [14].

The symptoms presented depend on the localization of the cyst and the degree of compression of the local organs [15]. There have also been reports of primary hydatid cysts in various organs such as the heart [16], soft tissue [17], adrenal gland [18] and brain [19].

Symptoms of pleural hydatidosis are similar to those found in pleural effusions such as dyspnea, mediastinal shift and reduction in lung volume. Even so, up to 15% of cases can be asymptomatic [20]. However, these can also be present alongside chest pain and other signs of cardiac or vascular involvement or compression [21]. Hydatid disease is also a rare cause of recurrent acute pulmonary embolism. This complication may develop after invasion of the cardiovascular system or direct invasion of the inferior vena cava [22].

In diagnosing hydatid disease, the most important part is played by imaging studies. Standard chest radiography alongside computed tomography, not only helps in diagnosing the disease but it also plays a role in the planning of surgery. Some radiological signs are pathognomonic for hydatid disease such as the presence of daughter cysts, water-lily sign (for ruptured cysts), and serpent sign (ruptured and completely evacuated cysts) [23]. Other tests such as skin tests, complement fixation, blood eosinophil count and indirect hemagglutination tests can be used, but must be interpreted carefully as they have a tendency towards false-positive results [24]. In addition, even if an ultrasound would be very useful in diagnosing liver cysts, it is rarely used for cysts in the thorax, with the exception of a chest wall hydatid cyst [25,26].

However, information provided by performing an ultrasound in such cases might bring significant information, complementary to those reported by other imagistic studies such as computed tomography; moreover, it can be safely performed in pregnant patients in whom such lesions are suspected [27,28]. Therefore, in pleural hydatidosis ultrasound might reveal suggestive aspects such as complex cystic lesions presenting double line sign whenever collapsed membranes exist [27]. In this respect, ultrasound has been successfully added as part of the examination tools in cases in which intrathoracic extrapulmonary hydatidosis is suspected, Gursoy et al. reporting the successful use of this method in a case series of 14 patients [14].

Although fine needle aspiration is recommended in the diagnosing of primary liver hydatid cysts, due to the high risk of complications such as pneumothorax, haemoptysis, cyst rupture, anaphylaxis and dissemination, performing this procedure on cysts located in the lung is not advised [29]. If we were to ignore the risks associated with this procedure and perform it, the most likely result is the discovery of hydatid material in the aspirated liquid.

However, in particular cases, puncture might orientate the diagnostic; therefore, in pregnant patients in whom the imagistic methods of evaluation are restricted due to the risk of fetal irradiation, performing an ultrasound examination and an ultrasound guided puncture might serve as an important diagnostic tool; therefore, in the case presented by a French study group and published in 2011 the authors reported the case of a 23-year-old patient who was investigated during the 22nd week of pregnancy for chest pain and dyspnea in association with pneumothorax; after performing puncture and establishing the diagnostic, the patient was further submitted to surgery, the cystic mass being removed; meanwhile, two bronchial fistulas were identified which were successfully sutured while the remaining cavity was capitonnaged [28].

The most common preoperative complications that may occur in pleural or lung hydatid disease include empyema and pneumothorax [30], but there are rare cases where, due to the rupture of the cysts, the patient can suffer from anaphylactic shock. There have even been cases of patients presenting with tension pneumothorax caused by the rupture of a lung cyst [31]. Several representative studies with patients with pulmonary hydatid cysts concluded that the rupture of the cyst in the pleural cavity is the most severe complication of the disease, alongside anaphylactic shock [32].

The main treatment in hydatid disease consists of surgery followed by medical treatment with antiparasitic medication. For pulmonary hydatid disease, the most common surgical approach is by thoracotomy and resection of the cysts. However, when the disease is present bilateral a median sternotomy is preferable [24]. The main goal of surgery is complete removal of the germinative membrane after inactivation using a hypertonic saline solution [33]. Standard surgical procedure consists of cystectomy and capitonnage; however, there are cases when an extended resection is necessary when the surrounding tissue is diffusely involved or there is presence of local infection or giant cysts. In all cases, local contamination during surgery must be avoided using a hypertonic saline solution to inactivate the cysts. If this is not performed thoroughly the chances of a recurrent disease increase significantly [34]. There are however several authors that consider that capitonnage is not necessary after cystectomy and that only the bronchial openings should be sutured and the cavity should be left open, arguing that capitonnage causes lung disfigurement, prolongs the operating time and increasing morbidity [35,36]. For primary pleural hydatidosis, the same surgical principles apply when it comes to approach and case management. Removal of the cysts and pleural fluid after inactivation with hypertonic saline solution is mandatory, followed by chest drainage paired with Albendazole treatment [14].

Antiparasitic treatment plays a key role in the management of any form of hydatidosis. Most authors recommend treatment with one of two benzimidazole carbamates, Albendazole or Mebendazole, which are the only drugs that interrupt larval growth of Echinoccocus species. Some authors consider that preoperative antiparasitic treatment is very useful, especially in severe forms of illness, and that it would reduce the recurrence by up to three and a half times [37]. In our opinion, preoperative antiparasitic treatment is extremely useful, especially in severe cases with a high risk of recurrence and intraoperative dissemination, such as the case we have presented.

The most used treatment is with Albendazole, while Mebendazole is only used as an alternative due to its adverse effects. However, antiparasitic treatment is recommended as anti-infection therapy, while the main course of treatment remains surgical [38].

As it can be observed, primary pleural hydatidosis is a rare entity, only rare cases being reported so far; experience gathered at this moment is rather related to case reports or case series presenting cases of patients diagnosed with intrathoracic extrapulmonary hydatidosis in general. Data published so far regarding this pathological entity are summarized into Table 1.

## 4. Conclusions

There are very few cases of primary pleural hydatidosis reported in the literature. In our case, it was a diagnosis of exclusion for hydatid cysts within the pleural cavity, but with no obvious primary lesion that would explain the local contamination.

Cases of primary pleural hydatidosis are very rare but must be taken into consideration in patients from endemic regions with jobs that may have exposure to this parasite. Proper treatment, both surgical and antiparasitic medication, can lead to a full recovery and a low chance of recurrent disease.

Antiparasitic treatment plays a key role in the management of any form of hydatidosis; however, the main course of treatment is surgical.

## Figures and Tables

**Table 1:** Cases reported so far diagnosed with primary pleural hydatidosis.

Name, Year	Period of the Study	No. of Cases	Location of the Lesion	Surgical Treatment	Associated Resections (no. of Cases)	Medical Treatment	Postoperative Complications
Gursoy et al., 2009 [14]	2003–2007	14	Diaphragm Chest wall Mediastinum Pleura Pericardial cavity	Cystectomy Decortication Resection and repair of the adjacent organs	Costal resection-3 pericardial resection-1	Albendazole 10 mg/kg during the next 3 months postoperatively	None
Marghli et al., 2011 [28]	2011	1	Pleural hydatid cyst in a 23-year-old pregnant woman	Removal of the cyst, suture on the bronchial fistula, capitonnage	None	Not reported	Uneventful
Mardani et al., 2017 [13]	2017	1	Pleural hydatidosis in a 33-year-old woman	Removal of the cyst, complete lung expansion	None	Not reported	Uneventful
Tewari et al., 2009 [12]	2009	1	Pleural hydatidosis in a 28-year-old woman	Removal of the cyst	None	Albendazole	Uneventful
Rakower et al., 1964 [7]	1954–1964	3	Mediastinum Chest wall Pleura	Removal of the cysts	Pleural resection	Not reported	One patient died due to septic shock
